# Psychiatric disorders in offspring of childhood or adolescent central nervous system tumor survivors: a national cohort study

**DOI:** 10.1002/cam4.3591

**Published:** 2020-11-01

**Authors:** Wuqing Huang, Kristina Sundquist, Jan Sundquist, Jianguang Ji

**Affiliations:** ^1^ Center for Primary Health Care Research Lund University/Region Skåne Lund Sweden; ^2^ Department of Family Medicine and Community Health Department of Population Health Science and Policy Icahn School of Medicine at Mount Sinai New York NY USA; ^3^ Center for Community‐based Healthcare Research and Education (CoHRE) Department of Functional Pathology School of Medicine Shimane University Matsue Japan

**Keywords:** central nervous system tumor, epidemiology, offspring, psychiatric disorders, survivorship

## Abstract

**Background:**

Children experience a higher risk of psychiatric problems when their parents are diagnosed with cancer. However, the psychological effect among offspring who are born after parental cancer diagnosed in childhood or adolescence is unknown. We aimed to investigate the risk of psychiatric disorders in children of survivors with childhood or adolescent central nervous system (CNS) tumors.

**Methods:**

By combining several nationwide Swedish registers, we identified all children who had at least one parent previously diagnosed with CNS tumor below the age of 20. Five children without parental CNS tumor were randomly selected for the matching. Cox proportional hazards model was used to calculate hazard ratios (HRs) with 95% confidence interval (CI).

**Results:**

The incidence rate of psychiatric disorders was 8.46 per 1000 person‐years in children of CNS tumor survivors, whereas the rate was 7.47 in the matched comparisons, yielding an adjusted HR of 1.10 (95% CI = 0.94, 1.28). Boys of survivors had a higher risk of psychiatric disorders (adjusted HR = 1.29, 95% CI = 1.04, 1.59). The risk of the specific types of psychiatric disorders in children of tumor survivors was comparable with that in the matched comparisons, except for mental retardation. Children of survivors experienced 2.36 times higher risk of mental retardation (95% CI = 1.21, 4.58), mainly of mild mental retardation (adjusted HR = 2.99, 95% CI = 1.40, 6.38).

**Conclusion:**

Children of survivors with CNS tumor in early life did not experience a significantly increased risk of overall psychiatric disorders, with the exception of an elevated risk of mental retardation that was mainly mild.

## Graphical abstract

Children whose mother or father was ever diagnosed with central nervous system tumor below age of 20 didn't have a higher risk to experience mental diseases, however, they were at an increased risk of mild intellectual disability.

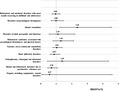

## 1 INTRODUCTION

A growing number of studies have examined the mental health of children of cancer patients, during their parents’ diagnosis and treatment.^1^, ^2^ These children often had a higher risk of psychiatric problems for multiple reasons, such as the decrease in physical and emotional availability of the parent, alteration of daily routines, shifting of household roles, and financial stress.^1^, ^2^ However, the mental health of children born after parental cancer in childhood and adolescence has been less studied.^3^ Existing evidence has indicated that individuals with a CNS tumor in early life have a higher risk of adverse long‐term neurologic and psychiatric outcomes due to primary and adjunct therapy, which could persist into adulthood.^4^, ^5^, ^6^, ^7^, ^8^, ^9^, ^10^ Furthermore, maternal or paternal exposure to stress or chemicals may induce epigenetic mutations, which are transgenerational and may affect their children's brain function and predisposition to psychiatric disorders.^11^, ^12^, ^13^, ^14^ Therefore, we hypothesized that a history of CNS tumor before adulthood might affect the mental health of children born subsequently. Central nervous system (CNS) tumor is one of the most frequently diagnosed malignancies among the younger population ^15^. With the development of modern cancer therapy and the associated improved survival rate, a greater number of patients with CNS tumor in early life can survive until adulthood and have children^16^, ^17^; it is thus imperative to examine various health outcomes, including the mental health, in the children of these CNS tumor survivors.

By linking several Swedish national registers, we aimed to explore the risk of psychiatric disorders in offspring of survivors with childhood or adolescent CNS tumor as compared to offspring without parental CNS tumor in childhood or adolescence, as well as the risk of the specific types of psychiatric disorders.

## 2 METHODS

### 2.1 Study population

We identified all singleton live births that occurred between 1973 and 2014 from the Swedish Medical Birth Register. This register was established to include childbirth information in Sweden from 1973 and onwards. The Swedish Multi‐generation Register allowed us to identify the parents of these children. By linking these parents to the Swedish Cancer Registry created in 1958,^18^ we obtained the information of CNS tumor diagnoses in these parents. We then extracted data concerning all children whose parent was ever diagnosed with a CNS tumor below the age of 20 and survived for at least 5 years after the cancer diagnosis. Five children, whose parents were not diagnosed with a CNS tumor, were randomly selected for matching to each child in the study group to generate the reference group, conditional on the same year of childbirth (continuous), the sex of offspring, maternal and paternal age at birth (continuous). We excluded children who were born within 1 year after parental diagnosis with a CNS tumor or died within 3 months after birth.

Every resident who lives in Sweden longer than 3 months is assigned a unique individual national identification number. To secure people's integrity, the identification number has been replaced with a serial number, which was used to link the registers used in this study.

### 2.2 Assessment of exposure and covariates

Details of parental diagnosis with CNS tumors were collected from the Swedish Cancer Registry, including maternal or paternal diagnosis, date at diagnosis, and histologic type of tumor. To investigate the effects of parental age at diagnosis, survivors diagnosed under the age of 15 were defined as childhood survivors, and those diagnosed between 15 and 19 were defined as adolescent survivors. The calendar year of parental diagnosis was divided into before or after 1990 to examine the potential influence of in vitro fertilization, which was first adopted in 1982 and very rare before 1990 in Sweden.^19^ To explore the histologic‐specific impact, we classified CNS tumors into the following types: astrocytoma, neurinoma, ependymoma, meningioma, hemangioma, medulloblastoma, and others.^16^, ^20^, ^21^ Parental highest education was retrieved from the Total Population Register established in 1960, modeled as 1‐9 years, 10‐11 years, and 12+ years. Parental diagnosis with psychiatric disorders was obtained from the National Patient Register (NPR). Preterm birth was defined as a live birth occurring at less than 37 full weeks of gestation, collected from the Swedish Medical Birth Register.

### 2.3 Assessment of outcomes

Diagnosis of psychiatric disorder in the offspring of CNS tumor survivors was retrieved from the NPR, which was established in 1964 and includes data with complete inpatient care since 1987 and data with specialized outpatient care since 2001. The International Classification of Disease (ICD) was used to classify different diseases in this register, and the 7th version was used before 1969, the 8th version from 1969 to 1986, the 9th version from 1989 to 1996, and the 10th version after 1996. We transformed the ICD‐7, ICD‐8, and ICD‐9 codes to ICD‐10 codes to ensure the consistency of the diagnoses. To explore the risk of specific types of psychiatric disorders, we classified the diagnoses into 10 main types of psychiatric disorders: Organic, including symptomatic, mental disorders (ICD10 codes: F00‐F09); Mental and behavioral disorders due to psychoactive substance use (ICD10 codes: F10‐F19); Schizophrenia, schizotypal and delusional disorders (ICD10 codes: F20‐F29); Mood [affective] disorders (ICD10 codes: F30‐F39); Neurotic, stress‐related and somatoform disorders (ICD10 codes: F40‐F48); Behavioral syndromes associated with psychological disturbances and physical factors (ICD10 codes: F50‐F59); Disorders of adult personality and behavior (ICD10 codes: F60‐F69); Mental retardation (ICD10 codes: F70‐F79); Disorders of psychological development (ICD10 codes: F80‐F89); Behavioral and emotional disorders with onset usually occurring in childhood and adolescence (ICD10 codes: F90‐F99). In addition, mental retardation was further categorized into mild type (F70) and others (F71‐F79: moderate, severe, profound, and others). Three criteria were used for mental retardation diagnosis according to the DSM‐5 (APA, 2013).^22^ The score of standardized IQ tests ranging from 50 to 69 indicates mild mental retardation.^23^

### 2.4 Statistical analysis

Cox proportional hazard models were used to calculate hazard ratios (HRs) and 95% confidence interval (CI). Follow‐up for psychiatric disorder began at the date of childbirth and ended at the date of the first‐time diagnosis of any psychiatric disorder, date of emigration, death, or end of the study (31 December 2015), whichever came first. Several covariates were taken into account in the multivariate analysis, including the year of childbirth, the sex of offspring, maternal and paternal age at birth, maternal and paternal highest education, maternal and paternal diagnosis with psychiatric disorders. Stratified analyses were further performed by comparing with matched comparisons to explore whether the impact of parental CNS tumor differed depending on the sex of the child, maternal or paternal tumor diagnosis, parental age at tumor diagnosis, year of tumor diagnosis, parental psychiatric disorders, preterm birth and time interval between parental tumor diagnosis and childbirth, which was categorized into 1‐10, 10‐20, >20 years.

We also investigated the risk of specific psychiatric disorders using Cox proportional hazard models. The follow‐up for the specific types of psychiatric disorders began at the date of childbirth and ended at the date of the first‐time diagnosis of the specific type of psychiatric disorder, date of emigration, death, or end of the study (31 December 2015), whichever came first. Analyses were stratified by sex of the child, maternal or paternal diagnosis, and parental age at diagnosis. In addition, as the cause of mild mental retardation is distinct from other mental retardation, we further explored the risk of mild mental retardation and other mental retardations separately.

## 3 RESULTS

We identified a total of 1364 children (633 girls and 731 boys) whose parents had been previously diagnosed with a CNS tumor below the age of 20. A total of 6820 children (3165 girls and 3655 boys) were selected as matched comparisons, as shown in Table 1,

**TABLE 1:** Sociodemographic characteristics among offspring of survivors with central nervous system (CNS) tumor and matched comparisons

Variables	Offspring of survivors		Matched comparisons	
	No. of individuals	No (%). of outcome	No. of individuals	No (%). of outcome
Overall	1364	189 (13.8)	6820	827 (12.1)
Sex of offspring				
Female	633	78 (12.3)	3165	399 (12.6)
Male	731	111 (15.2)	3655	428 (11.7)
Year of childbirth				
<2001	721	150 (20.8)	3605	653 (18.1)
≥2001	643	39 (6.1)	3215	174 (5.4)
Maternal age at birth				
<25	304	60 (19.7)	1520	252 (16.6)
25‐29	438	59 (13.5)	2190	275 (12.6)
≥30	622	70 (11.2)	3110	300 (9.6)
Paternal age at birth				
<30	530	82 (15.5)	2650	384 (14.5)
30‐34	447	61 (13.6)	2235	240 (10.7)
≥35	387	46 (11.9)	1935	203 (10.5)
Maternal highest education				
1‐9 y	123	26 (21.1)	685	84 (12.3)
10‐11 y	673	112 (16.6)	3145	436 (13.9)
>=12 y	568	51 (9.0)	2990	307 (10.3)
Paternal highest education				
1‐9 y	199	39 (19.6)	969	133 (13.7)
10‐11 y	673	99 (14.7)	3387	418 (12.3)
> =12 y	492	51 (10.4)	2464	276 (11.2)
Maternal diagnosis with mental diseases	280	56 (20.0)	1066	162 (15.2)
Paternal diagnosis with mental diseases	217	31 (14.3)	846	128 (15.1)
Parental tumor				
Maternal	720	104 (14.4)	3600	442 (12.3)
Paternal	644	85 (13.2)	3220	385 (12.0)
Parental age at tumor diagnosis				
Childhood	883	130 (14.7)	4415	553 (12.5)
Adolescence	481	59 (12.3)	2405	274 (11.4)
Year of parental tumor diagnosis				
<1990	1190	182 (15.3)	5950	775 (13.0)
≥1990	174	7 (4.0)	870	52 (6.0)
Histological type of parental tumor				
Astrocytoma	741	94 (12.7)	3705	465 (12.5)
Meningioma	41	9 (21.9)	205	31 (15.1)
Neurinoma	116	16 (13.8)	580	75 (12.9)
Ependymoma	82	13 (15.9)	410	44 (10.7)
Medulloblastoma	38	5 (13.2)	190	23 (12.1)
Haemangioma	47	8 (17.0)	235	21 (8.9)
Others	299	44 (14.7)	1495	168 (11.2)

**FIGURE 1:**
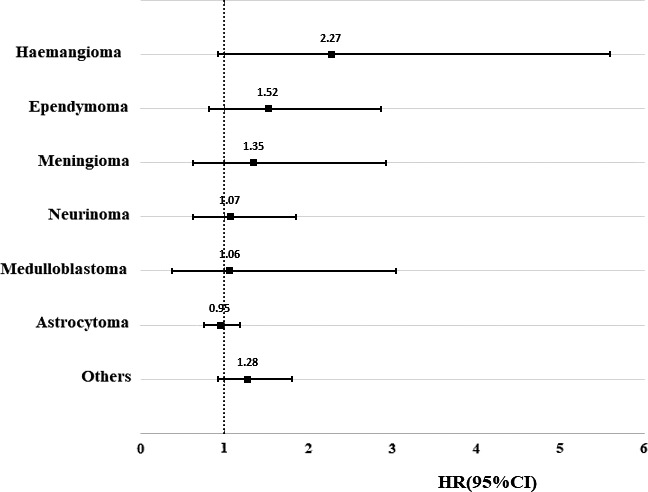
Hazard ratio of psychiatric disorders among offspring of survivors with central nervous system tumor compared with matched comparisons stratified by histologic type. HRs were adjusted for year of childbirth, sex of offspring, maternal and paternal age at birth, maternal and paternal highest education, maternal and paternal diagnosis with psychiatric disorders

As shown in Table 2, a total of 187 children of CNS tumor survivors developed a psychiatric disorder, yielding an incidence rate of 8.46 per 1000 person‐years, while the rate in the matched comparisons was 7.47 per 1000 person‐years. After adjusting for the confounding factors, there was no significant difference in the incidence rate of psychiatric disorders between children of cancer survivors and the matched comparisons (crude HR: 1.13, 95% CI: 0.96, 1.32; adjusted HR: 1.10, 95% CI: 0.94, 1.28). However, the risk in boys was significantly higher compared to the reference (adjusted HR: 1.29, 95% CI: 1.04, 1.59). Additional stratified analyses indicated a stronger association in children of female CNS tumor survivors, of childhood CNS survivors, and first‐born children after parental diagnosis but the associations were non‐significant. The HRs for the different histological types of parental tumors ranged from 0.95 (astrocytoma) to 2.27 (hemangioma). However, none of these were statistically significant (Figure 1).

**TABLE 2:** The hazard ratio of psychiatric disorders among offspring of survivors with central nervous system tumor compared with matched comparisons

Variables	Number of outcomes		Number of person‐years		IR / per 1000 person‐years		Crude HR	Adjusted HR^a^
	Survivors	Matched comparisons	Survivors	Matched comparisons	Survivors	Matched comparisons		
Overall	189	827	22332	110728	8.46	7.47	1.13 (0.96, 1.32)	1.10 (0.94, 1.28)
Sex of offspring								
Female	78	399	10182	50421	7.66	7.91	0.97 (0.76, 1.23)	0.90 (0.71, 1.15)
Male	111	428	12150	60307	9.14	7.10	1.28 (1.04, 1.58)	1.29 (1.04, 1.59)
Parental tumor								
Maternal	104	442	12147	59719	8.56	7.40	1.15 (0.93, 1.42)	1.11 (0.89, 1.37)
Paternal	85	385	10185	51009	8.35	7.55	1.11 (0.87, 1.40)	1.08 (0.85, 1.37)
Parental age at diagnosis								
Childhood	130	553	14519	71733	8.95	7.71	1.16 (0.95, 1.40)	1.11 (0.92, 1.35)
Adolescence	59	274	7812	38995	7.55	7.03	1.07 (0.81, 1.42)	1.05 (0.79, 1.39)
Year of parental diagnosis								
<1990	182	775	21204	105021	8.58	7.38	1.16 (0.99, 1.36)	1.13 (0.96, 1.32)
≥1990	7	52	1127	5707	6.21	9.11	0.71 (0.32, 1.57)	0.79 (0.36, 1.77)
Maternal diagnosis with mental diseases								
Yes	56	162	4227	16449	13.25	9.85	1.34 (0.99, 1.82)	1.29 (0.95, 1.76)
No	133	665	18104	94279	7.35	7.05	1.04 (0.86, 1.25)	1.03 (0.86, 1.25)
Paternal diagnosis with mental diseases								
Yes	31	128	3872	13698	8.01	9.34	0.82 (0.56, 1.22)	0.80 (0.54, 1.18)
No	158	699	18459	97030	8.56	7.20	1.19 (1.00, 1.41)	1.16 (0.98, 1.38)
Preterm birth								
Yes	19	42	1405	5158	13.53	8.14	1.72 (1.00, 2.97)	1.44 (0.81, 2.56)
No	170	785	20927	105570	8.12	7.44	1.09 (0.92, 1.28)	1.06 (0.90, 1.25)
Multiple pregnancies after diagnosis								
First child	109	469	13112	65057	8.31	7.21	1.14 (0.93, 1.41)	1.12 (0.91, 1.38)
Second or more	80	358	9220	45671	8.68	7.84	1.10 (0.86, 1.40)	1.06 (0.83, 1.36)
Time interval between parental diagnosis and childbirth								
1‐10	104	473	12609	62951	8.25	7.51	1.10 (0.89, 1.36)	1.06 (0.85, 1.31)
11‐20	53	243	6386	31467	8.30	7.72	1.06 (0.79, 1.44)	1.04 (0.78, 1.41)
≥21	32	111	3337	16310	9.59	6.81	1.39 (0.94, 2.07)	1.38 (0.92, 2.05)

Abbreviations: CNS, central nervous system; CI, confidence intervals; HR, hazard ratio; IR, incidence rates.

^a^ Adjusted for year of childbirth, sex of offspring, maternal and paternal age at birth, maternal and paternal highest education, maternal and paternal diagnosis with psychiatric disorders.

**FIGURE 2:**
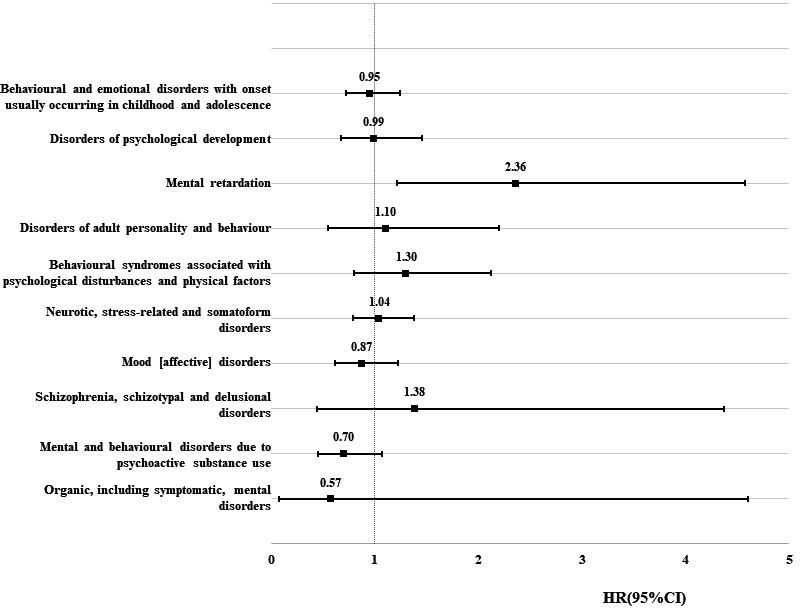
Hazard ratio of the specific type of psychiatric disorder among the offspring of survivors with central nervous system tumor compared with matched comparisons. CI, confidence intervals; HR, hazard ratio. HRs were adjusted for year of childbirth, sex of offspring, maternal and paternal age at birth, maternal and paternal highest education, maternal and paternal diagnosis with psychiatric disorders. (Data are shown in Supplementary Table S1)

Regarding the risk of specific types of psychiatric disorders, as shown in Figure 2 and Supplementary Table S1, the risk in children of tumor survivors was comparable with the matched comparisons, except for mental retardation. Children of survivors with a CNS tumor had a significantly elevated risk of mental retardation (crude HR: 2.29, 95% CI: 1.19, 4.42; adjusted HR: 2.36, 95% CI: 1.21,4.58), especially children of survivors with astrocytoma (adjusted HR: 2.93, 95% CI: 1.67,2.78). As shown in Table 3, the observed association seemed to be more pronounced in boys (adjusted HR:2.47), children of female survivors (adjusted HR: 2.91), and childhood tumor survivors (adjusted HR:2.71). The observed association seemed to be mainly contributed by a mild type of mental retardation (HR: 2.99, 95% CI: 1.40, 6.38).

**TABLE 3:** The hazard ratio of mental retardation among offspring of survivors with central nervous system tumor compared with matched comparisons

Variables	Number of outcome		Number of person‐years		IR / per 1000 person‐years		Crude HR	Adjusted HR^a^
	Survivors	Matched comparisons	Survivors	Matched comparisons	Survivors	Matched comparisons		
Overall	13	28	23464	115864	0.55	0.24	2.29 (1.19, 4.42)	2.36 (1.21, 4.58)
Sex of offspring								
Female	5	13	10656	52819	0.47	0.25	1.90 (0.68, 5.33)	2.32 (0.82, 6.62)
Male	8	15	12808	63045	0.62	0.24	2.62 (1.11, 6.18)	2.47 (1.04, 5.87)
Parental tumor								
Maternal	8	15	12734	62392	0.63	0.24	2.61 (1.11, 6.14)	2.91 (1.21, 7.01)
Paternal	5	13	10730	53472	0.47	0.24	1.90 (0.68, 5.34)	1.93 (0.68, 5.46)
Parental age at diagnosis								
Childhood	10	18	15296	75080	0.65	0.24	2.72 (1.26, 5.9)	2.71 (1.24, 5.94)
Adolescence	3	10	8168	40784	0.37	0.25	1.50 (0.41, 5.44)	1.83 (0.50, 6.77)
Type of mental retardation								
Mild	11	18	23834	119412	0.46	0.15	3.01 (1.42, 6.38)	2.99 (1.40, 6.38)
Others	2	10	23834	119412	0.08	0.08	0.99 (0.22, 4.50)	1.10 (0.24, 5.08)
Histological type of parental tumor								
Astrocytoma	9	15	12613	62205	0.71	0.24	2.95 (1.29, 6.75)	2.93 (1.27, 6.78)
Meningioma	1	0	943	4711	1.06	0.00	‐	‐
Ependymoma	1	0	1525	7628	0.66	0.00	‐	‐
Neurinoma	0	5	2234	11054	0.00	0.45	‐	‐
Medulloblastoma	0	1	708	3448	0.00	0.29	‐	‐
Haemangioma	0	0	626	3162	0.00	0.00	‐	‐
Others	2	7	4815	23656	0.42	0.30	1.40 (0.29, 6.75)	1.62 (0.33, 8.03)

Abbreviations: CNS, central nervous system; CI, confidence intervals; HR, hazard ratio; IR, incidence rates.

^a^ Adjusted for year of childbirth, sex of offspring, maternal and paternal age at birth, maternal and paternal highest education, maternal and paternal diagnosis with psychiatric disorders.

## 4 DISCUSSION

This retrospective cohort study is the first population‐based study to explore the mental health of children of survivors who were diagnosed with a CNS tumor before adulthood. We found that the overall mental health of these children was comparable with children of parents without a CNS tumor. However, children of tumor survivors were at 2.36 times higher risk of mental retardation. Mild mental retardation contributed mostly to increased risk (HR = 2.99). The elevated risk was more pronounced in boys and children of female or childhood survivors. Although the underlying mechanisms of the higher incidence of mental retardation in the offspring of these survivors are unclear, more clinical attention on these children may be needed as well as more school support.

Previous studies have demonstrated an increased risk of psychiatric problems among children of cancer patients diagnosed and treated for cancer in adulthood.^1^, ^2^ However, in terms of children born after a parental diagnosis with a CNS tumor in childhood and adolescence, we did not observe a higher burden of psychiatric disorders when compared with matched comparisons. The results are consistent with our previous study in the offspring of overall cancer survivors.^3^ However, boys of these survivors seemed to experience a slightly higher risk of psychiatric disorders.

The findings of an elevated risk of mental retardation, predominately mild mental retardation, is noteworthy, which is in line with our previous study that children of these survivors tended to get a poorer school performance.^20^ The causes behind the intellectual and learning disabilities in mental retardation are not completely clear. Genetics plays a critical role in severe and profound mental retardation, which often appears as part of syndromes, such as Down syndrome and fragile X syndrome.^24^ Mild mental retardation may be more likely to be linked to epigenetic changes and sociocultural and psychological causes.^24^, ^25^, ^26^, ^27^, ^28^, ^29^ The epigenetic changes might be induced by prenatal exposures (maternal alcohol use, drug use, malnutrition, and infection) and postnatal causes (whooping cough, measles, meningitis, and malnutrition).^25^, ^26^, ^27^, ^28^, ^29^ Sociocultural and psychological causes include poor environment, insufficient parent‐child interactions or early learning experiences.^25^, ^26^, ^27^, ^28^, ^29^ In this study, children of tumor survivors had a higher risk of mild mental retardation rather than a severe type thus indicating that epigenetic changes and sociocultural causes might have contributed to the observed findings while genetics may play a smaller role. Sociocultural and psychological problems are often tied to parental educational level and parental mental health. In the multivariate model, including parental educational level and parental diagnosis with psychiatric disorders, the association between parental CNS tumor and mild mental retardation remained, however, similar. This may indicate that there are other factors involved in the association, which calls for further investigations. Emerging evidence has suggested that parental exposure to radiation, chemicals, or stress might lead to transgenerational epigenetic programming of brain development in offspring.^11^, ^12^, ^13^, ^14^ Besides, parental tumors caused by germline neurofibromatosis type 1 (NF1) alterations may contribute to the observed association given that NF1 and other RAS‐driven brain tumor syndromes are known to cause mild behavioral and cognitive abnormalities in children.^30^ Additionally, tuberous sclerosis 1 (TSC1)/TSC2 mutations cause subependymal giant cell astrocytoma and are the main cause of autism‐like symptoms in children.^30^ Mild mental retardation might not be apparent before a poor academic performance is recognized in school.^26^ Results from the Carolina Abecedarian Project suggest that early intervention is beneficial for children to obtain better academic attainment, more employment opportunities, and fewer behavioral problems.^31^, ^32^ Therefore, survivors who were previously diagnosed with CNS tumor may be recommended to be observant of their children's intellectual development, especially adaptive functioning, such as skills of communication, self‐care, self‐direction, and academic skills.

We acknowledge several limitations in this study. A multicenter cohort study with a larger sample may be needed to confirm the observation. Furthermore, we were unable to examine the risk of psychiatric disorders in children associated with parental cancer treatments due to the lack of such information in our datasets. Several strengths should, however, be noted for this study. The external validity is warranted due to the nationwide coverage, the high quality of the registers, and the clinical diagnoses of psychiatric disorders. The matched cohort design helped to reduce the impact from several important covariates, such as the sex of the child, parental age at birth, and other potential confounders. Other covariates were further included in the multivariate model.

To sum up, children of survivors diagnosed with a CNS tumor during childhood or adolescence did not seem to have an increased risk of overall psychiatric disorders, but they had a significantly increased risk of mainly mild mental retardation. Intervention programs may be needed for these children to improve their future cognitive ability.

## CONFLICT OF INTEREST

None.

## AUTHOR CONTRIBUTIONS

Wuqing Huang, Jianguang Ji, Kristina Sundquist, and Jan Sundquist were involved in conception and design. Jianguang Ji, Kristina Sundquist, and Jan Sundquist were involved in financial support, administrative support, and provision of study material or patients. Kristina Sundquist and Jan Sundquist were involved in collection and assembly of data. Wuqing Huang was involved in data analysis and interpretation and manuscript writing. All authors were involved in final approval of manuscript and accountable for all aspects of the work.

## ETHICAL STATEMENT

The Ethics Committee at Lund University has approved (February 6, 2013) this nationwide cohort study (Dnr 2012/795). Written informed consent is not needed in Sweden for the register‐based study.

## Supporting information

Table S1

## Data Availability

Data cannot be shared publicly due to the confidentiality under the Swedish legislation. Registry‐based data are available from the appropriate Swedish authorities (the Swedish National Board of Health and Welfare (https://www.socialstyrelsen.se/en) and Statistics Sweden (https://www.scb.se/en), for researchers who meet the criteria for access to confidential data.
