# Supplementary material for: Psychiatric disorders in offspring of childhood or adolescent central nervous system tumor survivors: a national cohort study
Source: Cancer Med. 2020 Nov 1;10(2):675–83. doi: 10.1002/cam4.3591 (PMC7877351; doi:10.1002/cam4.3591)
Supplement: Supplementary file 1 — Table S1 [file CAM4-10-675-s001.pdf]

Supplementary Table 1 Hazard ratio of each specific type of psychiatric disorder among offspring of survivors with central nervous system tumor compared with matched comparisons.

**Variables**	**Number of outcome**		**Number of person-years**		**IR / per 1000 person-years**		**Crude HR (95%CI)**	**Adjusted HR (95%CI)[a]**
	**Survivors**	**Matched comparisons**	**Survivors**	**Matched comparisons**	**Survivors**	**Matched comparisons**		
**Specific disease**								
Organic, including symptomatic, mental disorders	1	8	23539	116038	0.04	0.07	0.61(0.08,4.91)	0.57(0.07,4.60)
Mental and behavioural disorders due to psychoactive substance use	24	162	23382	114929	1.03	1.41	0.72(0.47,1.11)	0.70(0.45,1.07)
Schizophrenia, schizotypal and delusional disorders	4	12	23532	115980	0.17	0.10	1.65(0.53,5.09)	1.38(0.44,4.37)
Mood [affective] disorders	39	209	23352	114961	1.67	1.82	0.91(0.65,1.28)	0.87(0.62,1.22)
Neurotic, stress-related and somatoform disorders	60	273	23250	114741	2.58	2.38	1.08(0.82,1.43)	1.04(0.79,1.38)
Behavioural syndromes associated with psychological disturbances and physical factors	21	74	23396	115490	0.90	0.64	1.40(0.86,2.27)	1.30(0.80,2.12)
Disorders of adult personality and behaviour	10	43	23479	115825	0.43	0.37	1.14(0.58,2.28)	1.10(0.55,2.20)
Mental retardation	13	28	23464	115864	0.55	0.24	2.29(1.19,4.42)	2.36(1.21,4.58)
Disorders of psychological development	30	148	23345	115238	1.29	1.28	1.00(0.68,1.48)	0.99(0.67,1.46)
Behavioural and emotional disorders with onset usually occurring in childhood and adolescence	62	316	23198	114270	2.67	2.77	0.96(0.73,1.27)	0.95(0.72,1.24)

[a] Adjusted for year of childbirth, gender of offspring, maternal and paternal age at birth, maternal and paternal highest education, maternal and paternal diagnosis with psychiatric disorders.
